# The effects of postmenopausal hormone therapy on social activity, partner relationship, and sexual life – experience from the EPHT trial

**DOI:** 10.1186/1472-6874-9-16

**Published:** 2009-06-08

**Authors:** Elina Hemminki, Piret Veerus, Heti Pisarev, Sirpa-Liisa Hovi, Päivi Topo, Helle Karro

**Affiliations:** 1 Health Services and Policy Research, National Institute for Health and Welfare (THL), PO Box 30, 00271 Helsinki, Finland; 2 Department of Epidemiology and Biostatistics, National Institute for Health Development (TAI), Hiiu 42, 11619 Tallinn, Estonia; 3 Department of Public Health, Tartu University, Ravila 19, 50411 Tartu, Estonia; 4 Finnish Office for Health Technology Assessment (Finohta), National Institute for Health and Welfare (THL), PO Box 30, 00271 Helsinki, Finland; 5 Health Services and Policy Research, National Institute for Health and Welfare (THL), PO Box 30, 00271 Helsinki, Finland; 6 Department of obstetrics and gynecology, University of Tartu, Lossi 36, 51003 Tartu, Estonia

## Abstract

**Background:**

With the exception of sexual functioning and weight, social and behavioural effects of postmenopausal hormone therapy (HT) have not been reported from trials. This paper reports such results from the EPHT-trial in Estonia.

**Methods:**

A randomized trial, with a blind and non-blind sub-trial in Estonia. From 1999–2001, 1778 women were recruited. The mean follow-up was 3.6 years. Women's experiences were asked in the first and final study year by mailed questionnaires (74 and 81% response rates). Comparisons of the groups were made by cross-tabulation and logistic regression, adjusting for age.

**Results:**

There were no differences between the HT and non-HT groups in regard to being employed, the extent of social involvement or marital status or opinions on aging. There was no difference in the frequency of free-time exercise, or overweight. Some of the indicators suggested less sexual inactivity, but the differences were small.

**Conclusion:**

In a trial setting, postmenopausal hormone therapy did not influence work or social involvement or health behaviour.

**Trial registration:**

ISRCTN35338757

## Background

The health effects on diseases, symptoms and well-being of postmenopausal hormone therapy (HT) have been a focus of research for good reason. In lay-thinking HT is often perceived to lead to better work and social capacity and family harmony [1,2]. With the exception of sexual functioning and weight (as a proxy for health habits), non-health benefits, such as better work capacity and social involvement have not been studied in trials, devoid of selection bias, and the data of surveys are also limited. Surveys of both physicians and women have focused on the level of HT use and the medical reasons for use; non-medical reasons have been rarely asked. When it has been asked, some physicians [3-7] and women [5,8-17] report they prescribe or use HT to improve sexual functioning or to delay aging, or at least they believe it would.

In addition to studying the effects of HT on health and health services in our trial in Estonia [18], we also wanted to study the effects on work, social activities, partner relationships, sexual life and physical activity, as well as on women's views on aging. This information was asked in annually mailed questionnaires. We report cross-sectional comparisons of the treatment groups on the first year and last year, because these surveys contained most of the relevant questions to this study.

## Methods

Postmenopausal women aged 50–64 and living in Tallinn (the capital of Estonia), Tartu, and in two counties surrounding these towns were asked to participate in a postmenopausal hormone therapy trial [18]. Potentially eligible women were randomized into four trial groups: 1) blind hormone therapy group, 2) blind placebo group, 3) non-blind hormone therapy group, 4) non-treatment group. Randomization occurred before mailing the invitation for the recruitment visit in order to study the impact of blinding on recruitment. The non-blind sub-trial was designed to study the impact of hormone therapy on health service utilization.

Altogether 1001 women were recruited into the non-blind sub-trial and 777 in the blind sub-trial at three clinical centres between January 1999 and December 2001. Details of the trial design, flow-chart, randomization, eligibility criteria, recruitment, and clinical follow-up of participants are described elsewhere [18]. Trial treatment was stopped by May 2004. The mean follow-up period for trial participants was 3.6 years. All participants gave written informed consent. The trial design was approved by the Tallinn Committee of Medical Ethics and the Research Ethics Committee of University Clinic of Tampere. The trial is registered as ISRCTN35338757 (ISRCTN register. ).

All participants were mailed annual questionnaires that included questions about their working status, weight and physical exercising, social activities, marital status, and sexual life. The first year survey and the final survey (filled in on average 3.6 years (range 2.2–4.9 years) after inclusion into the study) contained the most relevant questions to this study. The response rates in the surveys were 74 and 81%. There was no difference between the trial groups in the response rates to annual questionnaires. The key questions (translated into English) are given in Appendix 1. Some of them were made for other purposes than studying the social and behavioural effects. The first-year questionnaire included an instrument "Women's Health Questionnaire" [19], with some questions on aging and sex being relevant to our analyses; they are indicated by the abbreviation WHQ in brackets in the results. The proportion of women not having a partner was derived from questions on partner satisfaction or from questions on importance of sexual life (see Appendix).

Results on adherence and the reasons for non-adherence have been reported separately [20]. The proportion of women in the hormone therapy groups who took over 20% of their assigned trial medication was 83% at the end of the first trial year, and 64% by the end of the third year (same for the blind and non-blind group). Throughout the trial, about 90% of women in the non-treatment group and 95% in the placebo group did not start hormone therapy.

Because knowing or not knowing that one is taking active hormone therapy may be important for social and behavioural effects, the results are given separately as well as combined for the non-blind and blind sub-trials. Differences between proportions were tested by the Chi-square test. P-values of less than 0.05 were considered statistically significant. Women in the HT and no-HT groups (combining blind and non-blind sub-trials) were compared using age-adjusted logistic regression models. Statistical analyses were preformed with Intercooled Stata 9.2 (for contingency tables) and R 2.4.0 – A Language and Environment (for logistic regression); no-HT group was used as the reference.

The women's baseline characteristics were taken from the recruitment questionnaire, which was carried out on average 7.0 ± 4.4 months before the women were included into the trial, Table 1. The women in the HT and non-HT groups were very similar to each other. However, in the blind sub-trial the women in the placebo group were somewhat older, and we have age-adjusted the combined results.

**Table 1:** Description of the background characteristics of the women in the four groups^1^

Background characteristics	Blind sub-trial		Non-blind sub-trial	
	
	HT	Placebo	HT	Non-treatment
(n)	(n = 404)	(n = 373)	(n = 494)	(n = 507)

Age				

mean (SD)	58.4 (3.9)	59.0 (3.9)	58.6 (4.0)	58.9 (4.0)

50–54, %	24	20	25	21

55–59, %	39	34	36	35

60+, %	37	46	38	44

Education, years, %^2^				

<10	11	11	10	12

10–11	56	58	56	55

12+	32	31	35	32

Work status				

Employed	68	69	70	67

Retired	29	28	27	30

Other^3^	3	3	3	3

Marital status^4^				

Married	62	60	64	62

Widow	14	16	13	15

Divorced	18	18	17	17

Single	6	6	5	5

Births, mean (SD)	1.9 (1.0)	1.8 (0.9)	1.7 (1.0)	1.7 (1.0)

Age at menopause, mean (SD)	50.3 (3.7)	50.3 (3.9)	50.1 (3.7)	50.5 (3.8)

Height, cm, mean (SD)	164 (5.4)	163 (5.4)	164 (5.1)	164 (5.2)

BMI, kg/m^2^				

mean (SD)	26.9 (4.7)	26.9 (4.2)	27.2 (4.5)	26.9 (4.6)

25–29, %	39	42	39	40

30+, %	22	21	25	21

Current smoker, %	16	14	13	16

^1 ^From the recruitment questionnaire (before inclusion into the trial)

^2 ^No information = 0.2

^3 ^Working at home, unemployed, no information

^4 ^No information: 0.6%

Years of education were derived from the level: <10 years include preliminary and basic; 10–11 years) include secondary and vocational; 12+ years include higher and scientific degrees. Age at menopause was calculated according to the date of birth and the date of the last menstrual period as reported in the recruitment questionnaire. Body mass index (BMI) was calculated as weight in kilograms divided by square of height in meters.

To check the sensitiveness of our questions, we also did a logistic regression analysis on the age at recruitment. All outcome variables in which one would expect a decrease with age (being employed, being retired, psychically or physically exhausted, lives alone, exercise less frequently, no partner, no sex partner and other questions on own sexual activity) did actually show an association.

The study protocol was approved by the Tallinn Committee of Medical Ethics, Estonia, January 22, 1998, and by the Research Ethics Committee of University Clinic of Tampere, Finland, October 12, 1995.

## Results

There was no difference between the groups in regard to proportion of women being employed in the first and final surveys, Table 2.

**Table 2:** Work and social activity in the four groups

	Blind sub-trial		Non-blind sub-trial		HT vs. no HT^1^
	
	HT	Placebo	HT	None-treatment	OR 95% CI
1^st ^year (n)	(n = 306)	(n = 271)	(n = 364)	(n = 382)	

Employed^2^, %	62	62	63	63	0.89 (0.70–1.14)

Retired^2^, %	30	32	31	31	1.12 (0.86–1.46)

Final survey (n)	(n = 319)	(n = 301)	(n = 398)	(n = 428)	

Employed^3^, %	51	57	57	52	0.87 (0.69–1.09)

Retired^3^, %	37	35	35	37	1.12 (0.87–1.43)

No close friends, %	5.0	5.3	5.5	5.1	0.97 (0.61–1.55)

No neighbors visited, %	6.0	7.0	6.0	9.1	0.73 (0.49–1.10)

Does not belong to any group or club, %	66	65	64	66	0.92 (0.74–1.15)

Lives alone, %	19	22	21	22	0.92 (0.71–1.21)

^1 ^Age-adjusted odds ratios (95% confidence intervals), combining the blind and non-blind sub-trial, no-HT group is the reference.

^2 ^No information: 0.76%. The rest were working at home, unemployed or something else.

^3 ^No information: 0.35%.

In the final survey, the extent of social involvement was asked with a series of questions on number of friends, participation in groups or clubs and contacts with friends and family. Table 2 gives some examples of the indicators. In addition to those, the mean and median number of close friends and number of neighbours visited were calculated. None of the measures used showed differences between the groups.

There was no difference between the groups in regard to marital status (Table 3). There were no statistically significant differences between the groups in regard to having a partner or a sex partner.

**Table 3:** Marital status and partner relationships^1 ^in the four groups

	Blind sub-trial		Non-blind sub-trial		HT vs. no HT^6^
	
	HT, %	Placebo, %	HT, %	None-treatment, %	OR (95% CI)
1^st ^year					

Marital status^2^					

Married/cohabiting	63	59	63	64	1.01 (0.81–1.27)

Divorced	15	16	16	14	1.04 (0.77–1.41)

Single	8	6	6	6	1.21 (0.77–1.91)

No partner^3^	34	37	34	34	0.98 (0.78–1.23)

Final year					

Marital status^4^					

Married/cohabiting	60	57	61	59	1.06 (0.86–1.31)

Divorced	15	16	17	17	0.95 (0.72–1.26)

Single	7	5	6	5	1.20 (0.77–1.88)

No partner^5^	34	38	33	37	0.87 (0.70–1.09)

^1 ^For the denominators, See Table 3.

^2 ^The rest were widows (15%) or no information (0.8%).

^3 ^Partner refers to a man you a living with; from the question: "Which kind of views your partner has taken of your participation in this trial?"

^4 ^The rest were widows (18%) or no information (0.1%).

^5 ^From the question: "Are you satisfied with your partner as a friend?"

^6 ^Age-adjusted odds ratios (95% confidence intervals), combining the blind and non-blind arms, no-HT group is the reference.

In the first-year survey, sex and sexual relationships were the subject of several different questions. The women in the different groups were relatively similar in regard to having had sexual intercourse in the past year or being sexually active, Table 4. In the blind sub-trial the other indicators suggested somewhat more sexual inactivity or disinterest in the placebo group than in the HT-group. In the non-blind sub-trial, only responses to the question on sexual activity showed a difference, and even that was statistically non-significant. In the final survey, the proportions of women who had not had intercourse in the past year were somewhat higher in the non-HT groups (Table 4, combined analysis). Proportions of women whose partners had sexual difficulties or who were not satisfied with their partner as a lover were similar in all groups.

**Table 4:** Sexual life, experiences and views^1 ^in the four groups

	Blind sub-trial		Non-blind sub-trial		HT vs. no HT^9^
	
	HT, %	Placebo, %	HT, %	None-treatment, %	OR (95% CI)
1^st ^year					

No intercourse past year^2^	29	30	27	25	1.11 (0.87–1.42)

Not sexually active^3^	59	65	52	59	0.82 (0.67–1.00)

Satisfied with sexual relationship^4^	25	18^10^	19	21	1.11 (0.85–1.43)

Lost interest in sex^5^	39	45	41	44	0.91 (0.75–1.12)

Sex not important, in general^6^	29	21^11^	28	27	1.17 (0.91–1.50)

Final survey					

No intercourse past year^2^	45	52	47	52	0.77 (0.62–0.95)

Partner has difficulties^7^	18	17	16	15	1.03 (0.78–1.37)

Not satisfied with partner as a lover^8^	13	10	11	12	1.24 (0.93–1.65)

^1 ^For the denominators, see Table 3.

^2 ^From the question: "During the past 12 months I have had pains during intercourse"; no information 1.7% (1^st ^year) and 3.3% (final).

^3 ^From the question: "I am satisfied with my current sexual relationship"; includes no information group.

^4 ^From the question: "I am satisfied with my current sexual relationship", definitely; no partner or no information: 58% (WHQ).

^5 ^From the question: "I have lost interest in sexual activity", definitely (WHQ).

^6 ^From the question: "In my age, sexual life is not so important any more", totally agree, (Q49).

^7 ^From the question: "Does your partner experience difficulty in sexual performance?", rather much; no partner or no information: 49%.

^8 ^From the question: "Are you satisfied with your partner as a lover?"; not at all; no partner or no information: 46%.

^9 ^Age-adjusted odds ratios (95% confidence intervals), combining the blind and non-blind arms, non-HT group is the reference.

^10 ^P-value = 0.05.

^11 ^P-value = 0.04.

In the first-year survey painful intercourse in the past year was somewhat more common in the placebo and non-treatment groups than in the HT-groups, both in the blind sub-trial (5% vs. 8%) and the non-blind sub-trial (6% vs. 8%), but these differences were not statistically significant. In the final survey, there was no difference between the groups.

Weight (calculated as BMI) and the frequency of free-time exercise were asked in all annual surveys. We use them here as a proxy of physical activity. In the blind sub-trial, somewhat more women in the HT-group reported exercising than in the placebo group, both in the first and the final survey, Table 5. In the non-blind sub-trial, the findings were the opposite, but the differences between the groups were small and not statistically significant. Also the proportion of women in the final survey who reported being rather often or (almost) always psychically exhausted (32–37%) or physically exhausted (33–38%) were similar.

**Table 5:** Physical activity: exercise during free-time and body-mass-index (BMI)^1 ^in the four groups

	Blind sub-trial		Non-blind sub-trial		HT vs. no HT^6^
	
	HT, %	Placebo, %	HT, %	Placebo, %	OR (95% CI)
Exercise					

1^st ^year^2^					

Little/none	20	25	25	24	0.88 (0.68–1.14)

(Rather) much	42	34^7^	33	37	1.08 (0.86–1.35)

Final survey^3^					

Little/none	27	35^8^	32	35	0.78 (0.62–0.97)

(Rather) much	33	26	27	30	1.10 (0.88–1.39)

BMI					

1^st ^year^4^					

25–29	41	38	39	40	1.01 (0.81–1.27)

≥ 30	23	21	25	23	1.12 (0.86–1.45)

Final survey^5^					

25–29	38	39	39	43	0.91 (0.73–1.13)

≥ 30	24	24	27	22	1.16 (0.91–1.48)

^1 ^For the denominators, see Table 3.

^2 ^No information = 0.45%. The rest answered "some".

^3 ^No information = 1.1%. The rest answered "some".

^4 ^No information = 1.9%.

^5 ^No information = 2.7%.

^6 ^Age-adjusted odds ratios (95% confidence intervals), combining the blind and non-blind arms, non-HT group is the reference.

^7 ^P-value = 0.04.

^8 ^P-value = 0.03.

In the first-year and final survey, body mass index (BMI) did not vary between the groups, whether it was measured by proportion of overweight or obese (Table 5) or mean BMI (data not shown).

The first-year questionnaire included three questions related to aging, with no difference between the groups in regard to them, Table 6. In the final survey, women were asked "do you find it difficult becoming old". In the blind sub-trial, the proportion of women who said "rather or very much" was somewhat higher in the placebo group than the HT-group. In the non-blind sub-trial no difference between the groups was found.

**Table 6:** Opinions on own ageing in the four groups

	Blind sub-trial		Non-blind sub-trial		HT vs. no HT^5^
	
	HT, %	Placebo, %	HT, %	None-treatment, %	OR (95% CI)
1^st ^year					

Worried about growing old^1^	8.5	10	9.9	8.1	1.02 (0.70–1.48)

Happy with looks^2^	28	24	29	28	1.11 (0.87–1.41)

Husband blames climactericum^3^	35	35	29	32	0.94 (0.74–1.19)

Final year					

Becoming old difficult^4^	18	25	24	21	0.94 (0.73–1.21)

^1 ^From the question: "I worry about growing old", definitely; no information 3.9%, WHQ

^2 ^From the question: "I am happy with the way I look", definitely; no information 3.8%, WHQ.

^3 ^From the question: "My partner thinks my problems are due to climacteric", totally agree; no information 2.0%.

^4 ^From the question: "Do you find it difficult becoming old?" Rather much or very much; no information 1.8%.

^5 ^Age-adjusted odds ratios (95% confidence intervals), combining the blind and non-blind arms, non-HT group is the reference.

^6 ^P-value = 0.02.

## Discussion and Conclusion

In a trial setting, we found that postmenopausal hormone therapy had very little impact on employment status, social involvement or partner relationships. There was a marginal positive effect on sexual life. Taking into account our previous results of no effect on the quality of life [21], the value of HT should be judged on its symptom-relieving effects and disease effects; the latter have been mainly negative (see e.g. [22,23]).

However, three methodological reservations should be considered in regard to our results. First, our indicators were rough, and some were from questions originally made to measure other things. Secondly, the response rate was good for a survey, but still a large proportion of women did not respond. Thirdly, the exposure was weak as many women stopped taking their prescribed trial medication.

The two first-mentioned reservations are not likely to be important. Our analysis by age showed the indicators to be sensitive to age effects. Even though we do not know whether the non-respondents were similar in the different groups, the equal size of the non-respondents suggests so. The low exposure remains a potential problem, and one could argue that with higher exposure a greater effect could have been found.

We found no previous trials on the effect of HT on working status or social relationships. The small improvement for sexual indicators is in accordance with previous literature. Available trials suggest that HT, especially oestrogen, improves genital symptoms and consequently, sexual functioning (for reviews, see [24,25]). Nevertheless, the impact on sexual desire is less clear [24,25].

Among many women, loss of interest in sex and low satisfaction increase after menopause. This was found in this trial, as well as in previous population-based surveys [26-29]. Various factors influence sexuality [30]. In this trial, one third of the women did not have a partner, and others reported that the partner had sexual problems. But many women reported sexual problems related to the woman herself or the relationship. Thus, our results that suggested a weak positive effect of HT on sexual life are theoretically interesting, and warrant further studies on HT and sexuality. It would be important to tease out which are due to physical effects, such as dry vagina – easily corrected with lubricants – and which are due to psychological effects.

We had two indicators to measure heath behaviour: exercise during free-time and weight/BMI. Our results showed no impact of HT on these. However, weight and BMI may reflect metabolic effects rather than behavioural changes. The effects of HT on weight in previous trials have been inconsistent, varying from increasing to decreasing weight and no impact [31,32,24]. According to surveys, some women discontinue HT because of weight gain that they believed to be due to HT [14].

Our motive to study these non-medical effects in our trial was based on lay-beliefs we had faced both in literature and observed. Our hypothesis before the trial was that the non-medical effects of HT would be minimal – if any. Thus we did not systematically study potential biological pathways. It is likely that proponents of HT for social benefits assume the effects to rise through the increase in general well-being as well as through specific hormone effects, "aestrogen being necessary for a woman to be whole" (Watkins). Our trial did not support these beliefs. There was a weak indication of positive effects on sexuality, but the weakness of impact and negative disease effects of HT are likely to prevent HT being used as a sexual therapy. Further analysis of existing HT trials in regard to social effects would be welcome.

## Competing interests

The authors declare that they have no competing interests.

## Authors' contributions

EH designed the analysis and wrote the first draft, PV was the field coordinator, participated in the analysis and commented the manuscript, HP did the analysis and commented the manuscript, S-LH was the trial coordinator and commented the manuscript, PT commented the manuscript, and HK commented the manuscript. All authors read and approved the final manuscript. The EPHT-group as a whole was responsible for the design of the study and the field work.

## Appendix

The key questions (source questionnaire) used in the study.

• Your education? 1. Preliminary, 2. Basic, 3. Secondary, 4. Vocational, 5. Higher, 6. Scientific degree (recruitment)

• When was Your last period? Year/month (recruitment)

• Your height: _____ cm (recruitment)

• Your present weight: _____ kg (recruitment and annual)

• Do/did You smoke? 1. No, 2. Yes. I do, 3. Yes, I did earlier (recruitment)

• How much have you exercised during your free-time within past 3 months? 1. Not at all, 2. A little, 3. Somewhat, 4. Rather much, 5. Much (annual)

• Are you currently in paid work? 1. No, I am retired, 2. No, I am unemployed/looking for a job, 3. Yes, I am in paid work outside home, 4. Yes. I am working at home, 5. Something else, specify _____ (recruitment, annual)

• Who is/are living with you in the same household? (You may choose more than one alternative) 1. I live alone, 2. Husband or cohabitant, 3. Child/children, 4. My own parent(s), 5. My husband's/cohabitant's parent(s), 6. Others, who _____ (first year)

• What is your current marital status? 1. Single, 2. Married, 3. Cohabitant, 4. Divorced, 5. Widow (recruitment, annual)

• About how many families in your neighbourhood are you well enough acquainted with, so that you visit each other? _____ families (final)

• About how many close friends do you have – people you feel at ease with and can talk with about what is on your mind? (You may include relatives.) (Enter number on line) _____ close friends (final)

• How active are you in these groups or clubs you belong to? (If you belong to many, just count those you feel closest to. If you do not belong to any, circle 4.) 1. Very active, attend most meetings, 2. Fairly active, attend fairly often, 3. Not active, belong to but hardly ever go, 4. Do not belong to any groups or clubs (final)

• Which kind of views has your partner (elukaaslane, the man you are living with) taken about your participation in this trial? 1. I do not (currently) have a partner, 2. He disagrees, 3. He agrees, 4. He does not care, 5. He does not know, 6. Something else _____ (first year)

• Are you satisfied with your partner (elukaaslane) as a friend? 1. Not at all, 2. A little, 3. Rather much, 4. Very much, 5. I have no partner (final)

• During the past 12 months I have had pains during intercourse? 1. No, 2. Yes, 3. I have had no intercourse (first year, final)

• I am satisfied with my current sexual relationship (please omit if not sexual active)? 1. Yes, definitely, 2. Yes, sometimes, 3. No, not much, 4. No, not at all (WHQ, first year)

• I have lost interest in sexual activity? 1. Yes, definitely, 2. Yes, sometimes, 3. No, not much, 4. No, not at all (WHQ, first year)

• In my age sexual life is not so important any more? 1. I do not have a husband/cohabitant, 2. I totally agree, 3. I somewhat agree, 4. I don't know, 5. I somewhat disagree, 6. I totally disagree (first year)

• If you are married or a cohabitant, what is your partner's age? _____ years (first year)

• I worry about growing old? 1. Yes, definitely, 2. Yes, sometimes, 3. No, not much, 4. No, not at all (WHQ, first year)

• I am happy with the way I look? 1. Yes, definitely, 2. Yes, sometimes, 3. No, not much, 4. No, not at all (WHQ, first year)

• My partner thinks that my problems are due to climacteric? 1. I totally agree, 2. I somewhat agree, 3. I don't know, 4. I somewhat disagree, 5. I totally disagree (first year)

Do you find it difficult becoming old? 1. Not at all, 2. A little, 3. Rather much, 4. Very much, 5. I do not know (final)

## Pre-publication history

The pre-publication history for this paper can be accessed here:


